# Identification S100A9 as a potential biomarker in neuroblastoma

**DOI:** 10.1007/s11033-021-06783-2

**Published:** 2021-10-24

**Authors:** Xian Chen, Yukun Xue, Jiao Feng, Qingwu Tian, Yunyuan Zhang, Qing Wang

**Affiliations:** 1 Department of Clinical Laboratory, The Affiliated Hospital of Qingdao University, Qingdao, 266003 Shandong China; 2 Department of Clinical Laboratory, Pingyi Hospital of Traditional Chinese Medicine, Linyi, 273300 Shandong China; 3 Department of Pathology, The Affiliated Hospital of Qingdao University, Qingdao, 266003 Shandong China

**Keywords:** Neuroblastoma, Bioinformatics analysis, S100A9, Metastasis, Biomarker

## Abstract

**Background:**

More than half of Neuroblastoma (NB) patients presented with distant metastases and the relapse of metastatic patients was up to 90%. It is urgent to explore a biomarker that could facilitate the prediction of metastasis in NB patients.

**Methods and results:**

In the present study, we systematically analyzed Gene Expression Omnibus datasets and focused on identifying the critical molecular networks and novel key hub genes implicated in NB metastasis. In total, 176 up-regulated and 19 down-regulated differentially expressed genes (DEGs) were identified. Based on these DEGs, a PPI network composed of 150 nodes and 452 interactions was established. Through PPI network identification combined with qRT-PCR, ELISA and IHC, S100A9 was screened as an outstanding gene. Furthermore, in vitro tumorigenesis assays demonstrated that S100A9 overexpression enhanced the proliferation, migration and invasion of NB cells.

**Conclusions:**

Taken together, our findings suggested that S100A9 could participate in NB tumorigenesis and progression. In addition, S100A9 has the potential to be used as a promising clinical biomarker in the prediction of NB metastasis.

## Introduction

Neuroblastoma (NB), the most aggressive form of solid tumor of infants, accounting for 15% of cancer deaths in children [1, 2]. Until now, the main preferred treatment choices still remain surgery, chemotherapy and radiotherapy, which inevitably lead to tremendous toxicity and drug resistance. For high-risk NB patients, the recurrence and progression ratio are about 50% [3]. Thus, it is urgent for us to identify new effective biomarker for early diagnosis, metastasis prediction and ideal therapeutic target for NB patients.

S100A9, which is also called calgranulin B or migration inhibitory factor-related protein 14 (MRP-14), belongs to the low-molecular-weight calcium-binding S100 protein family [4]. Evidence has shown that S100A9 protein is elevated in metastatic melanoma and prostate cancer [5, 6]. In these tumors, increased expression of S100A9 was correlated with tumorigenesis and poor differentiation. Although S100A9 have been studied in many types of cancers, the biological function in malignancies was still remains contradictory and poorly understood. For example, elevated S100A9 in malignant tissues were associated with significantly shorter cancer survival, while downregulated S100A9 is correlated with tumor proliferation, inflammation invasion and angiogenesis [7–13]. To our knowledge, this is the first research to investigate the effects of S100A9 in NB patients.

In the present study, bioinformatic analysis was performed based on the GEO database [14]. After DEGs screening and functional analysis, PPI net-work of DEGs were further analyzed. And we found that the expression of S100A9 is pretty high in metastatic NB patients. In addition, we investigated the biological functions of S100A9 in NB cell line. These results may help us identify new effective biomarker for early diagnosis, metastasis prediction and ideal therapeutic target for NB patients, and provide valuable biological information for further investigation of NB.

## Materials and methods

### Bioinformatic analysis

#### Data processing and analysis

The public microarray dataset GSE90121, which was obtained based on the Affymetrix GPL570 platform (Affymetrix Human Genome U133 Plus 2.0 Array), was downloaded from the Gene expression omnibus (GEO) database (http://www.ncbi.nlm.nih.gov/geo/) [14]. This dataset was deposited by David Kaplan et al., containing information from human NB SK-N-AS metastatic subpopulations isolated after in vivo selection, aimed to identify genomic signatures that regulate metastasis and candidate therapeutics for NB patients. A total of 16 samples, including 12 metastatic samples and four primary samples, were enrolled in the current dataset. Robust multi-array average (RMA) affy package of Bioconductor was used to adjust the raw data. The processed gene expression data was then filtered to include those probe sets with annotations which reference the new version annotation files. To identify DEGs, we used the Linear Models (Microarray Data package in Bioconductor) to compare the expression levels of genes between the metastatic group and the localized tumor group [15]. An adjusted p-value of < 0.05 and a |log2FC (fold change) | of ≥ 2 was used as the threshold.

### Functional enrichment analysis of DEGs

Database for annotation, visualization and integrated discovery (DAVID) integrates a set of functional enrichment tools to distinguish functional genes underlying diseases processing (http://david.abcc.ncifcrf.gov/) [16]. Gene ontology (GO) function and Kyoto Encyclopedia of Genes and Genomes (KEGG) pathway enrichment analyses of DEGs were performed based on DAVID. P value < 0.05 and count ≥ 2 was regarded as statistically significant differences.

### Protein–protein interaction network construction by STRING

We used the Search Tool for the Retrieval of Interacting Genes (STRING), an online tool and biological database for prediction of interactions between proteins, to construct the PPI network [17]. According to our analysis based on STRING, score (median confidence) > 0.15 was the standard of PPIs for DEGs selections. The Cytoscape software was used to visualize the PPI network [18]. The proteins that have many interaction partners, were defined as the hub proteins, constituting the extremely important nodes in the PPI network. To identify such hub proteins in the PPI network, we utilized six bioinformatic tools, namely Closeness, Degree, EPC, MNC, Radiality and Stress centrality. Sub-network analysis was then conducted to help us discover the outstanding genes.

### NB patients, tissue samples and cell lines

Primary tumor tissues were obtained from 9 NB patients with bone marrow metastasis and 10 NB patients without bone marrow metastasis who had undergone tumor resection surgery at the Affiliated Hospital of Qingdao University. None of the included patients was treated with chemotherapy, hormonal therapy or radiotherapy before the tumor resection surgery. Written informed consent was obtained from all the participants. The current research was conducted with the permission of the Medical Ethical Committee of Affiliated Hospital of Qingdao University (Qingdao, China).

Human NB cell line SH-SY5Y was kindly provided by Professor Xiao from the Guizhou Medical University. The cells were cultured in Dulbecco’s modified Eagle’s medium (DMEM) contained 10% fetal bovine serum (FBS, Hyclone, USA) under the conditions of 37 °C, 5% CO_2_.

### Quantitative real-time PCR

The expression of *TAC1*, *PTGS2* and *FGF1*was examined by quantitative real-time reverse transcription polymerase chain reaction (qRT-PCR). The collected tissues were immediately frozen at − 80 °C after surgery. Total RNA was extracted from cancer tissues by TRIzol reagent (Invitrogen Life Technologies). Then the extracted RNA was transcribed into cDNA using random primers and analyzed with an ABI 7000 Real-Time PCR System (Applied Biosystems). PCR primers were as following: TAC1 primers: (forward) 5′-TGA TCT GAA TTA CTG GTC CGA CT-3′ and (reverse) 5′-TCC GGC AGT TCC TCC TTG A-3′; PTGS2 primers: (forward) 5′-TAA GTG CGA TTG TAC CCG GAC-3′ and (reverse) 5′-TTT GTA GCC ATA GTC AGC ATT GT-3′; FGF1 primers: (forward) 5′-CTC CCG AAG GAT TAA ACG ACG-3′ and (reverse) 5′-GTC AGT GCT GCC TGA ATG CT-3′; GAPDH primers (forward) 5′-CAG CGA CAC CCA CTC CTC-3′ and (reverse) 5′-TGA GGT CCA CCA CCC TGT-3′.Reactions were performed in triplicate using SYBR Green master mix (TaKaRa, Japan) and normalized to GAPDH mRNA level using the ΔΔCt method.

### NB serum samples and ELISA

After blood samples centrifuged at 3000 × *g* for 10 min at 4 °C, the serum samples were aliquoted and stored at− 80 °C until further processing. To quantify levels of S100A9 in serum, ELISA was performed as previously described [19]. By using human S100A9 (JYM0539Hu, JYM, China) ELISA kits, S100A9 in serum of the NB patients were detected according to the manufacturer’s recommended procedure.

### IHC staining

In brief, the formalin fixed, paraffin-embedded tissues sections were deparaffinized, rehydrated and boiled in 0.01 M citrate buffer for 10 min, then incubated with 0.3% H_2_O_2_ in methanol to block endogenous peroxidase activity. Then the sections were incubated with the anti-S100A9 antibody (Cell Signaling Technology, USA), followed by incubation with secondary antibody tagged with the peroxidase enzyme for 30 min at room temperature, finally visualized with 0.05% DAB (3,3′-diaminobenzidine) until the desired brown reaction product was obtained. All slides were observed under an OLYMPUS BX41 Microscope, and representative photographs were taken.

### Construction of plasmids and establishment of stably transfected cells

SBI-piggyBac vector, GST-S100A9, were kindly provided by Professor T.C. He from the University of Chicago. To construct an S100A9 overexpression plasmid, the complete coding sequence of human S100A9 gene was subcloned into the SBI-piggyBac vector. For S100A9 silencing, siRNAs targeting human S100A9 with the sequences of 5′- GCA AGA CGA TGA CTT GCA A -3′ and 5′- TTG CAA GTC ATC GTC TTG C -3′ were synthesized and assembled into the SBI-piggyBac vector, resulting in SBI-siS100A9. After transfecting SH-SY-5Y NB cells with the constructed plasmids, the stably transfected cells were selected by incubation with puromycin for one week. The stable transfected cell lines, namely Control (SH-SY5Y transfected with SBI empty vector), S100A9 (SH-SY5Y transfected with SBI-S100A9) and siS100A9 (SH-SY5Y transfected with SBI-siS100A9) briefly.

### Cell viability assay

The viability of SH-SY5Y cells was assessed by 3-(4,5-dimethylthiazol-2-yl)-2, 5-diphe-nyltrazolium bromide (MTT) assay. Briefly, stably transfected SH-SY5Y cells were seeded in 96-well plates (1000 cells/well). The cells were incubated in DMEM supplemented with 1% FBS for 24, 48, 72, 96 and 120 h, then incubated with MTT reagent (Progema, Madison, WI, USA, 20 µL/well) for another 4 h at 37 °C to allow the formation of formazan. After that, 100 µL of dimethyl sulfoxide was added into the cell culture medium for another 10-min incubation at room temperature. At last, in every day of the next five days, a microplate reader (Bio-rad, iMark) was used to measure the absorbance at 492 nm of each well. Each sample included three independent replicates.

### Colony formation assay

Exponentially growing stably transfected SH-SY5Y cells were seeded at a low density (100 cells/well) in cell culture medium containing 1% FBS in 6-well plates. The cells were allowed to grow for about 10 days to form colonies. The culture medium was refreshed every 3–4 days. Crystal violet was used to stained the colonies. Colony numbers from 3 wells were used to calculated the average colony number.

### Scratch wound healing assay

The scratch wound healing assay was performed as described previously [20, 21]. Briefly, stably transfected SH-SY5Y cells were seeded in 6-well plates and grown to ~ 90% confluency. Then, sterile micro-pipette tips were used to scratch the monolayer formed by SH-SY5Y cells to create a wound. After that, the medium (DMEM containing 1% FBS) was refreshed every day to remove the floating cells. Bright field microscopy was used to monitor the wound healing status at 24 h, 48 h, and 72 h after the wound was created. Each assay was repeated three times. ImageJ software was used to calculate the wound healing ratio.

### Transwell invasion assay

A chamber coated with non-type I-collagen (Millipore, USA) was used for the transwell assay. The upper chamber coated with ECM gel (Sigma, USA) was filled with 400µLof serum-free DMEM and seeded with exponentially growing stably transfected SH-SY5Y cells (1 × 10^4^ cells). The lower chamber was filled with 500µL of DMEM supplemented with 20% FBS, which served as a chemoattractant. After 24 h of incubation, the cells migrated across the transwell membrane were dried, fixed with methanol, and then stained with hematoxylin–eosin (H and E). Cotton swabs were used to remove the cells on the upper surface of the transwell membrane. At last, an inverted microscope (× 100 magnification) was used to count the number of cells migrated across the transwell membrane. Five randomly-selected fields were examined to obtain the mean value of the number of cells migrated across the transwell membrane. The experiment was repeated three times.

### Statistical analysis

All data are presented as means ± standard deviations. T test was performed in the GraphPad Prism software to determine the statistical significance of differences between groups. A P value of less than 0.05 was considered statistically significant.

## Results

### Enrichment analyses of DEGs

**Fig. 1:**
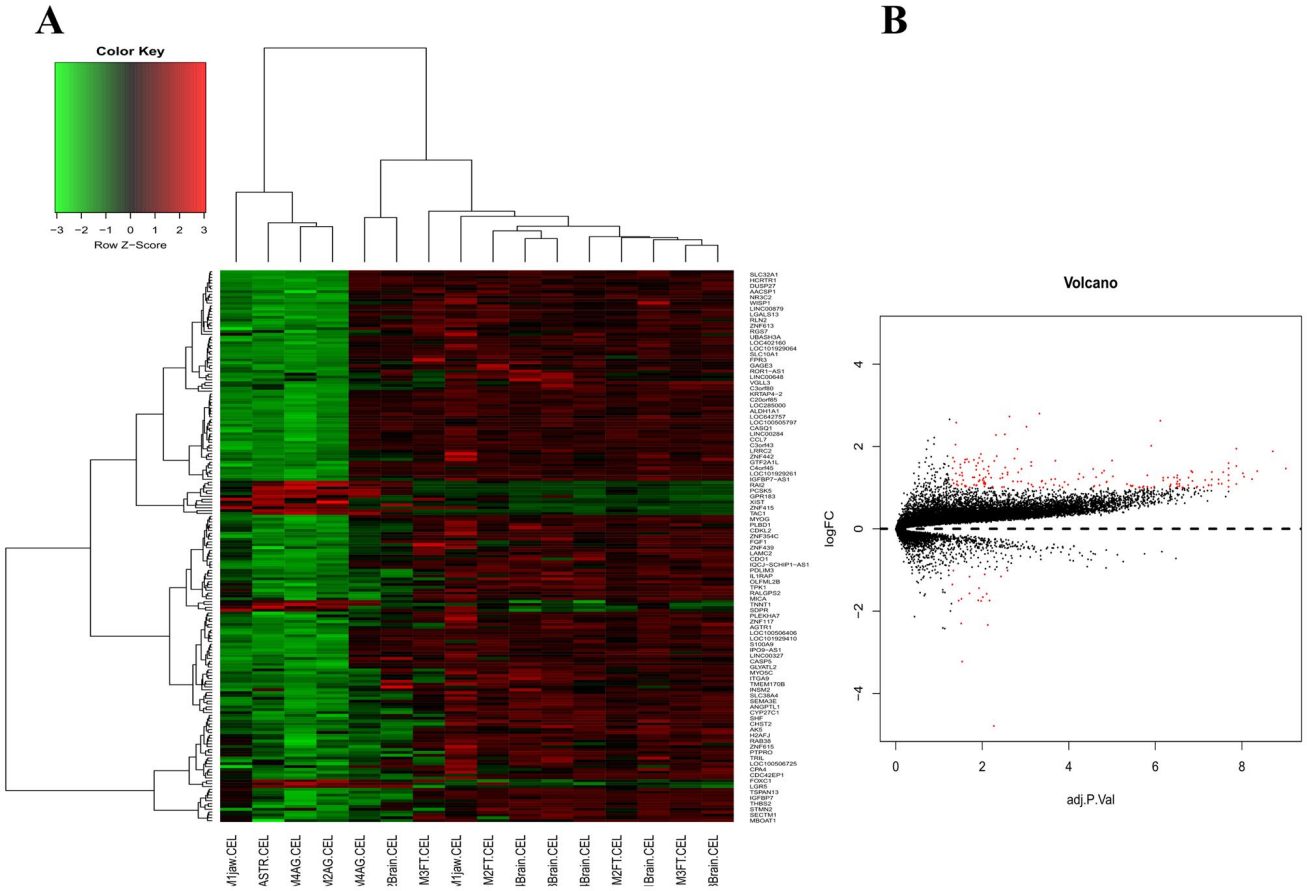
**A** Heat map for the differentially expressed genes (DEGs). **B** Volcano plots reflecting number, significance and reliability of differentially expressed mRNA in NB. The red dots indicate dysregulated mRNAs

In general, 176 up-regulated DEGs and 19 down-regulated DEGs were identified based on the cut-off criteria (p-value < 0.05 and count ≥ 2), which were used to generate a heatmap for the NB patients with and without metastasis (Fig. 1A). Functional enrichment analysis revealed that the top 18 mostly enriched GO terms and top two mostly enriched KEGG pathways were associated with regulation of nucleosome assembly, innate immune response in mucosa, extracellular matrix organization, angiogenesis and innate immune response, etc. (Table 1). In addition, volcano plot was able to quick identify the expression changes within the gene sets by combination of statistical tests (adjusted p-value) and magnitude of change (Fig. 1B).

**Table 1:** The tops enriched GO terms of the differentially expressed genes

Category	ID	Term	p-value	Gene count	Genes
BP	GO:0001558	Regulation of cell growth	3.13E-04	6	NANOS1, NRG3, IGFBP2, AGTR1, IGFBP7, WISP1
	GO:0006954	Inflammatory response	5.07E-04	11	CCL7, TRIL, C3AR1, FPR3, IL1RAP, CDO1, PTGS2, TAC1, HDAC9, S100A9, CHST2
	GO:0007204	Positive regulation of cytosolic calcium ion concentration	0.00316801	6	AGTR1, C3AR1, ADM, FPR3, LPAR3, TAC1
	GO:0030335	Positive regulation of cell migration	0.011814659	6	CCL7, GPNMB, CDH13, SEMA3E, LAMC2, FGF1
	GO:0051384	Response to glucocorticoid	0.012309797	4	IGFBP2, ADM, CDO1, PTGS2
	GO:0,007,200	Phospholipase C-activating G-protein coupled receptor signaling pathway	0.012827699	4	HTR1D, AGTR1, C3AR1, FPR3
	GO:0051482	Positive regulation of cytosolic calcium ion concentration involved in phospholipase C-activating G-	0.018038462	3	GPR65, AGTR1, LPAR3
	GO:0008285	Negative regulation of cell proliferation	0.027623627	8	MYOG, KAT2B, GPNMB, CDH13, IGFBP7,ADM,PTGS2,MYO16
	GO:0030819	Positive regulation of cAMP biosynthetic process	0.035152385	3	GPR65, RLN2, ADM
	GO:0007267	Cell–cell Signaling	0.040239268	6	CCL7, ADM, TAC1, PCSK5, S100A9, WISP1
CC	GO:0043005	Neuron projection	0.007369421	7	SLC32A1, CASP5, PTPRO, STMN2,CDH13,PTGS2,POU4F1
	GO:0005615	Extracellular space	0.007817435	19	GDF10, IGFBP2, SECTM1, ADM, LAMC2, SEMA3E, FGF1, PCSK5, WISP1, CCL7, NRG3, PLBD1, CDH13, IGFBP7, TAC1, S100A9, ANGPTL1, MICA, CPA4
	GO:0005576	Extracellular region	0.011007958	21	OLFML2B, IGFBP2, PSG1, ADM, LAMC2, SEMA3E, IL1RAP, FGF1, THBS2, FBLN2, APOLD1, CCL7, NRG3, RLN2, FDCSP, IGFBP7, CCDC3, VIP, TAC1, S100A9, CHGB
	GO:0005887	Integral component of plasma membrane	0.012602605	19	GABRB1, VIPR1, SLC10A1, GPR65, PTPRO, HTR1D, LPAR3, FPR3, IL1RAP, HCRTR1, TSPAN13, GPNMB, NRG3, GPR183, C3AR1, AGTR1, LGR5, MICA, SLC38A4
	GO:0005901	Caveola	0.079588673	3	SDPR, CDH13, PTGS2
MF	GO:0008201	Heparin binding	0.007960505	6	CCL7, GPNMB, LAMC2, THBS2, FGF1, WISP1
	GO:0003705	Transcription factor activity, RNA polymerase II distal enhancer sequence-specific binding	0.014352101	4	MYOG, FOXC1, POU4F1, VGLL3
	GO:0005179	Hormone activity	0.03603451	4	RLN2, ADM, VIP, CHGB

Category	ID	Term	Gene symbol	p-value
KEGG_PATHWAY	hsa04080	Neuroactive ligand-receptor interaction	GABRB1, VIPR1, HTR1D, AGTR1, C3AR1, FPR3, LPAR3, HCRTR1	9.46E-04
	hsa04512	ECM-receptor interaction	LAMC2, THBS2, ITGA9	0.090573261

### PPI network analysis

**Fig. 2:**
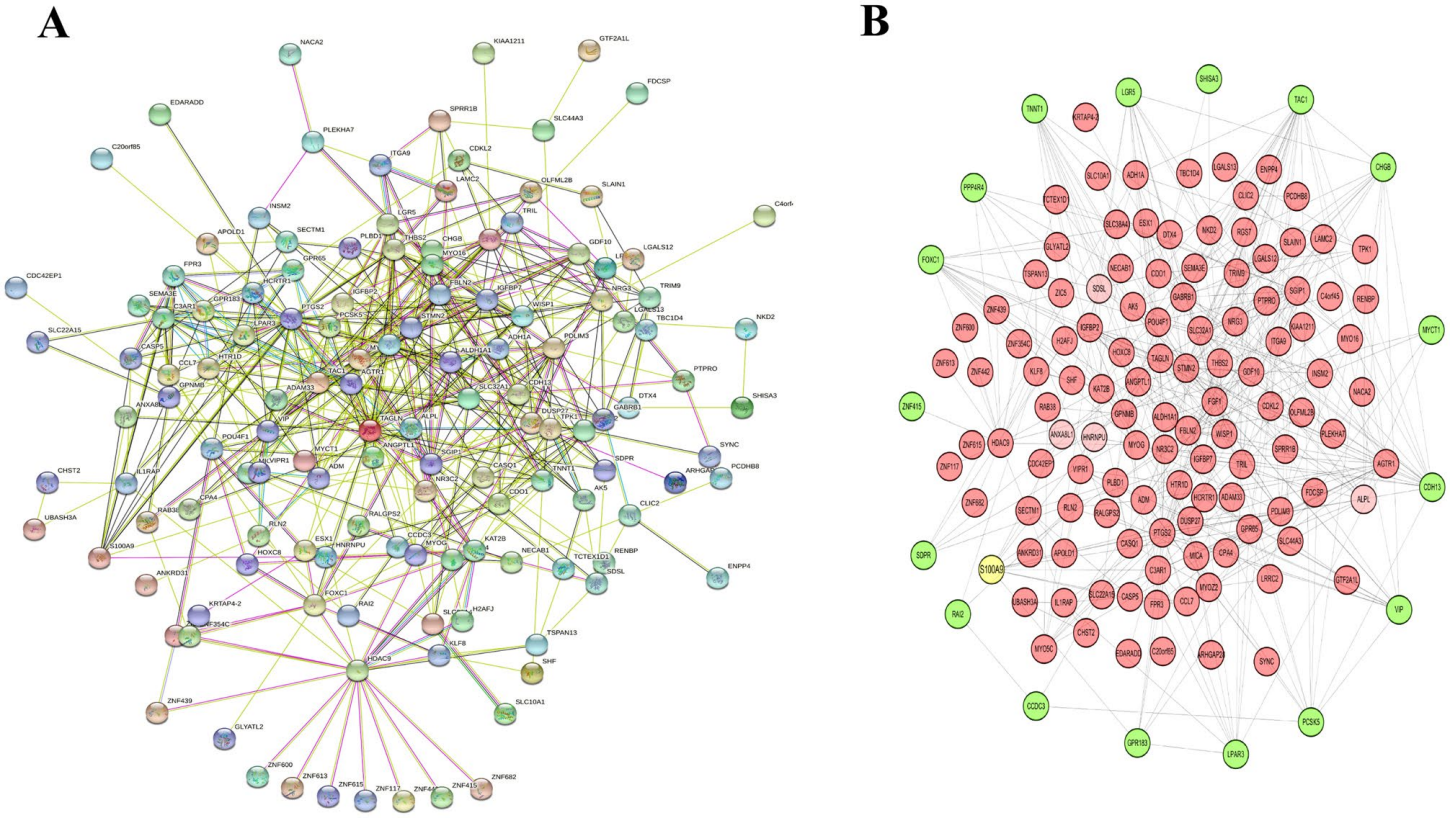
**A** Protein-protein interaction network constructed with the dysregulated differentially expressed genes. **B** PPI network was visualized by Cystoscope software

To identify the potential biomarkers that predict metastasis in NB patients, the DEGs were delineated to construct a PPI network. A DEG with a combined score (median confidence) > 0.15 was regarded as a significantly differentially expressed gene. The Cystoscope software was used to visualize the PPI network. As shown in Fig. 2A, the PPI network constructed by STRING is composed of 150 nodes and 452 interactions. The top 10 hub proteins in the PPI network determined by Closeness, Degree, EPC, MNC, Radiality and Stress centrality are listed in Table 2. The results showed that *TAC1*, *PTGS2* and *FGF1*were the most outstanding genes and maybe play an important role in the NB metastasis. Sub-network analysis suggested that S100A9 was the outstanding hub protein (Fig. 2B).

**Table 2:** Top 10 genes that evaluated by betweenness, BottleNeck, Closeness, Clustering coefficient, Degree, DMNC, EcCentricity, EPC, MCC, MNC, Radiality and Stress centrality respectively in the protein–protein interaction network

Gene name	Clinenms centrality	MNC, Radiality and stress centrality respectively in the protein–protein interaction network								Gene name	Stress centrality
		Gene name	Degree centrality	Gene name	EPC centrality	Gene name	MNC centrality	Gene name	Radiality centrality		
HDAC9	70.66667	5LC22A15	25	TRIM9	22.245	TAC1	25	TAC1	4.87121	PTGS2	7892
PTGS2	70.08333	ZNF43P	25	GPR183	22.121	STMN2	23	FGF1	4.86344	HDAC9	7010
TAC1	69.75	URASH3A	23	AK5	22.046	SLC32A1	23	PTGS2	4.84091	TAC1	6490
KAT2B	64.41667	CHST2	20	CASQ1	19.554	RGS7	18	TAGLN	4.79545	FGF1	6136
FOXC1	66.25	SEMA3E	19	WISP1	19.156	PTGS2	17	CDH13	4.7803	FOXC1	4312
FGF1	65.83333	HCRTR 1	18	RGS7	19.142	FGF1	17	AGTR1	4.72727	STMN2	4228
STMN2	65.75	GPR183	17	NRG3	18.906	CHGB	16	STMN2	4.7197	KAT2B	4132
MYOG	64.41667	FPR3	17	ANGPTL1	18.77	IGFBP7	14	MYOG	4.69697	CDH13	4034
CDH13	63.45	CCL7	17	SEMA3E	18.362	THBS2	14	KAT2B	4.68939	TAGLN	3990
TAGLN	62.83333	NRG3	16 15	GPR65	17.728	NRG3	13	SLC32A1	4.68182	AGTR1	3838

### Validation the outstanding genes in NB patients

**Fig. 3:**
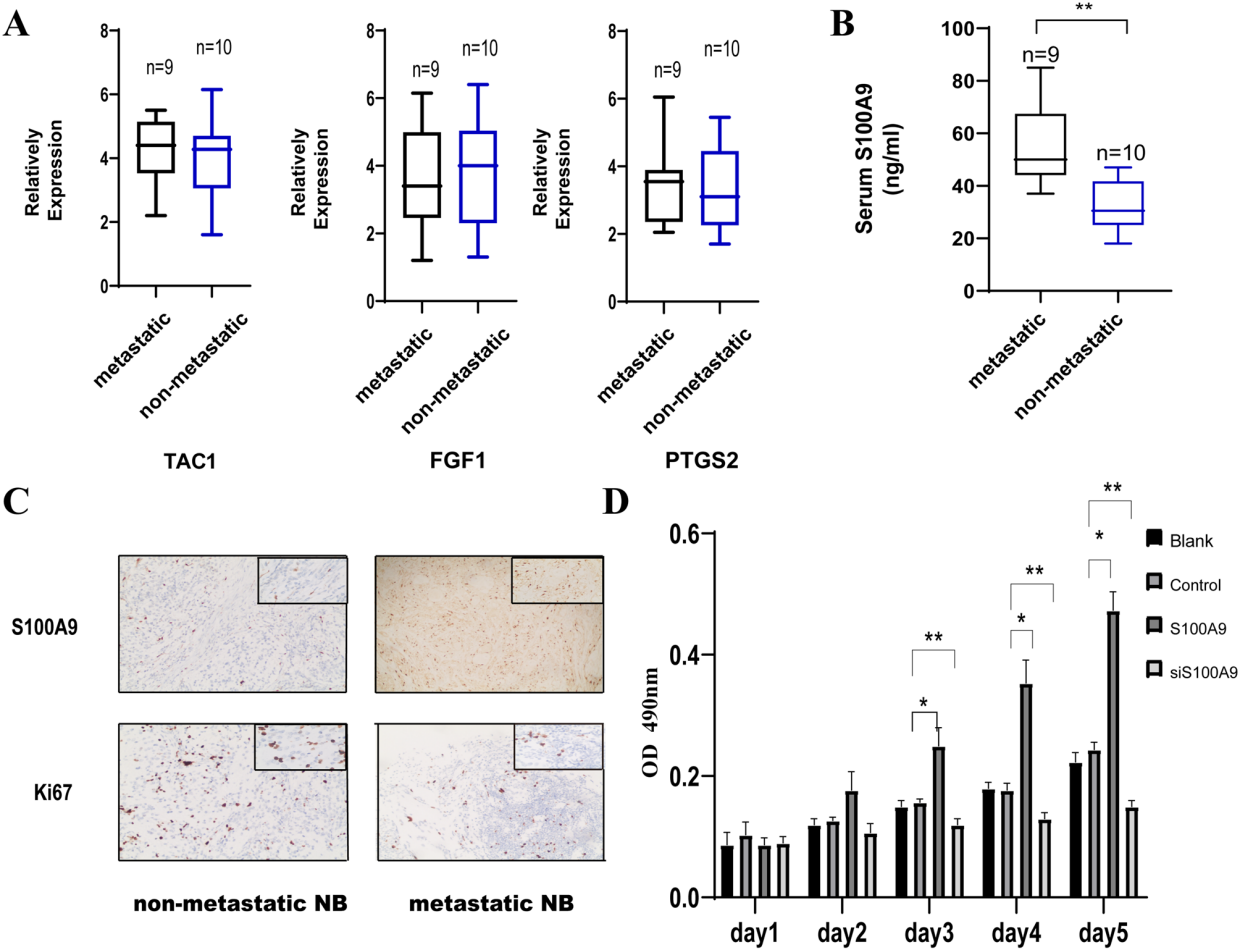
**A** qRT-PCR analysis for the expression of hub genes. **B** ELISA analysis for serum levels of S100A9 in metastatic (n=9) and non-metastatic NB (n=10) patients. **C** Representative IHC staining for S100A9 in tissue sections from metastatic and non-metastatic NB patients. **p < 0.01. **D** MTT analysis for Blank/Control/S100A9/siS100A9 SH-SY-5Y cells for sequential 5 days. *p<0.05, **p < 0.01.

To verify the expression of *TAC1*, *PTGS2* and *FGF1* in microarray data, qPCR was performed to detect the expression of those three genes. The qRT-PCR results showed that the expression of three genes were no significant differences between the 9 NB patients with bone marrow metastasis and 10 NB patients without bone marrow metastasis (Fig. 3A). Next, ELISA and IHC were performed to detect the protein levels of S100A9 in NB patients (9 NB patients with bone marrow metastasis and 10 NB patients without bone marrow metastasis).The serum levels of S100A9 were higher from NB patients with bone marrow metastasis than without bone marrow metastasis (Fig. 3B). Further, IHC staining was also used to examined the expression of S100A9 from NB patients’ tissues with and without bone marrow metastasis (Fig. 3C). The results showed that the expression levels of S100A9 were significantly higher in NB patients with bone marrow metastasis than NB patients without bone marrow metastasis.

### S100A9 overexpression promoted the proliferation, migration and invasion of NB cells

**Fig. 4:**
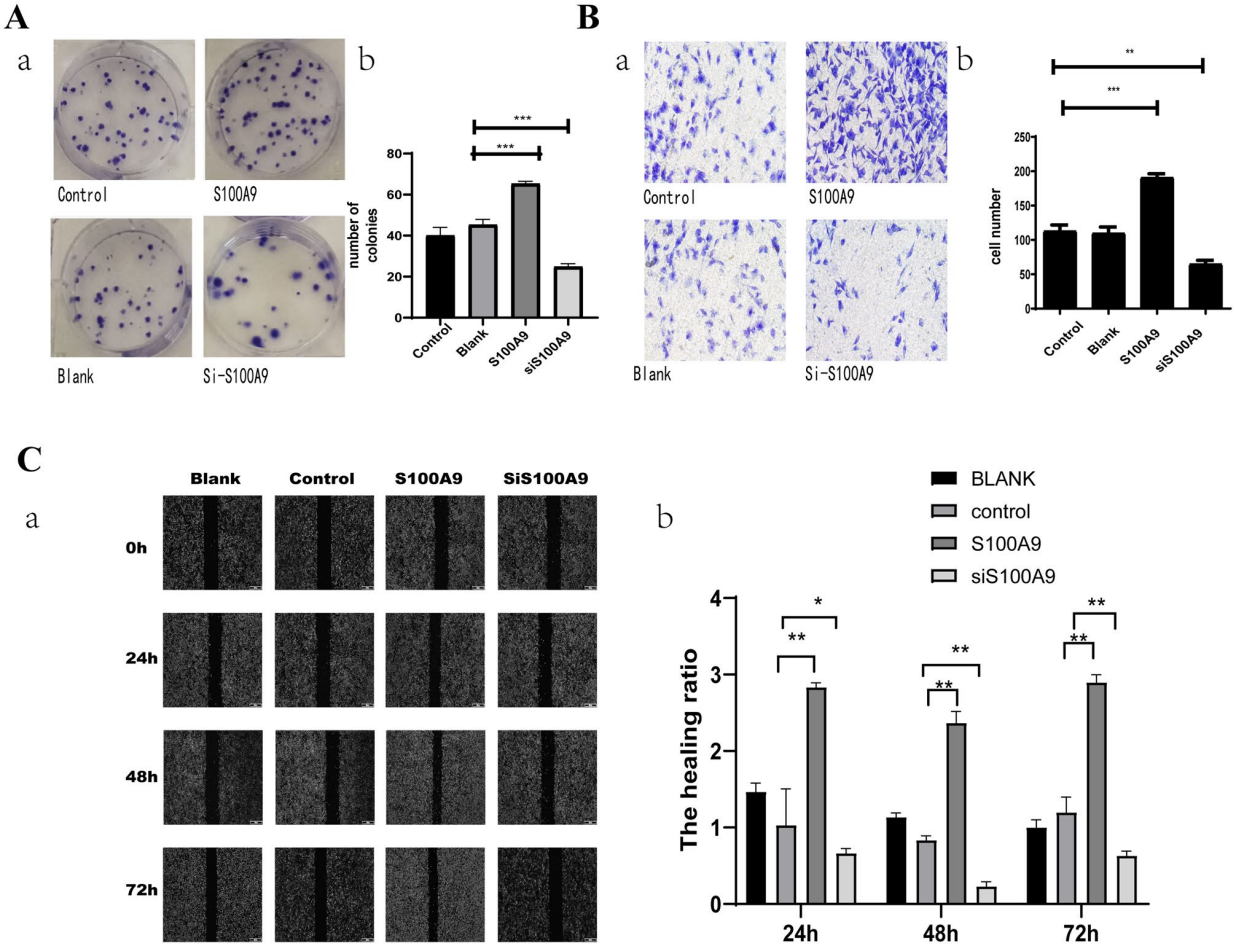
**A** a. Colony formation assay for Blank/Control/S100A9/siS100A9 SH-SY-5Y cells. b. The representative images of transmembrane cells are shown in the right panel. The mean numbers of transmembrane cells ± SD per microscopic field of three independent experiments are quantified in the right panel. **p < 0.01, ***p < 0.001. **B** a.Transwell invasion assay for Blank/Control/S100A9/siS100A9 SH-SY-5Y cells for 24 h. b.The representative images of transmembrane cells are shown in the right panel. The mean numbers of transmembrane cells ± SD per microscopic field of three independent experiments are quantified in the right panel. Magnification, ×100, **p < 0.01, ***p < 0.001. **C** a.Wound healing assay for Blank/Control/S100A9/siS100A9 SH-SY-5Y cells for 72 h. b.The incision width of different sites was measured and average healing rate was calculated and shown in the right panel. *p < 0.05, **p < 0.01

To investigate the effects of *S100A9* on the proliferation of NB cells, the coding sequence of human *S100A9* gene expressed in GST-S100A9 vector was subcloned into the SBI-piggyBac plasmid to overexpress *S100A9* in SH-SY5Y cells. According to the MTT assay results, the SH-SY5Y cells overexpressing *S100A9* (defined as the S100A9 group) exhibited higher proliferation ability than the SH-SY5Y cells transfected with empty vector (defined as the control group) at days 3, 4, and 5 (p < 0.05). The S100A9-knockdown SH-SY5Y cells (defined as the siS100A9 group) exhibited significantly slower proliferation when compared with the control group at days 3, 4, and 5 (p < 0.001) (Fig. 4A). Colony formation assay showed that the S100A9 group formed more colonies compared with the control group. Quantitatively, the number of colonies formed in the S100A9 group was approximately double than that of the control group (Fig. 4B). These results indicated that *S100A9* overexpression accelerated the proliferation of SH-SY5Y cells. In addition, as revealed by wound healing and transwell assays, the results exhibited that the migration and invasion of SH-SY5Y cells were significantly active by S100A9 (Fig. 4A and B).

## Discussion

High-throughput genome sequencing technologies have been widely used nowadays. Using these technologies, several research groups have identified genetic variations in human NB patients [22]. Among the identified genetic variations, MYCN amplification (32.1%), 11q loss (47.5%) and 17q gain (80.4%) were the most frequently observed ones (around 90% in total) in individuals with a high risk of developing NB [23]. Furthermore, two research groups found that recurrent genomic rearrangements affecting genomic regions close to the telomerase reverse transcriptase (*TERT*) gene locus led to significant transcriptional upregulation of *TERT* [24, 25]. However, even though great progresses have been made in understanding the genetic basis of NB tumor occurrence and development, effective biomarkers for prediction of metastasis in NB patients are still lacking.

In the current study, the GSE90121 dataset which was deposited by David Kaplan were downloaded and analyzed by bioinformatics method to identify potential crucial genes associated with NB metastasis. A total of 195 genes including 19 down-regulated and 176 up-regulated genes were obtained. Besides, the significantly enriched GO terms were mainly focused in regulation of cell growth, inflammatory response, positive regulation of cell migration. Hub genes of the regulatory network were then selected and conducted with PPI network module. Dysregulated *TAC1*, *PTGS2* and *FGF1* were the top three outstanding genes based on both six methods (Closeness, Degree, EPC, MNC, Radiality, and Stress centrality) evaluation. The expression levels of *TAC1*, *PTGS2* and *FGF1* in resected specimen of NB patients with or without metastasis were then validated by qRT-PCR. Although the expression of *TAC1*, *PTGS2* and *FGF1* were related with many kinds of tumorigenesis, such as non-small cell lung cancer, pancreatic ductal cancer, colorectal cancer, squamous cell carcinoma, gastric cancer and clear cell renal cell carcinoma[26–33], but these genes expression did not exhibit significant change as expected as the microarray results, indicating that these three genes may not the pivotal gene that participate in the metastasis of NB.

After go through the differentially expressed genes list and reviewing the relevant literatures, we found that S100A9 exhibits a broad range of biological functions involving in various cancer progression [34–36]. Characterized by calcium-binding EF hand motifs, S100 family comprise of more than 20 homologous proteins. Studies have revealed that S100A9 induced activation of NF-kB which participate in a broad range of intracellular and extracellular functions by regulating angiogenesis, tumor migration, wound healing, cell apoptosis, proliferation, differentiation, and inflammation [9, 37–42]. In addition, it has become increasingly evident that S100A9 acts as a potent amplifier of inflammation in tumor. S100A9 have been reported to be as Damage-associated molecular patterns (DAMPs) and involved in almost all aspects of cancer biology, such as proliferation, tumorigenesis, apoptosis, invasion, metastasis and angiogenesis. To our knowledge, fewer researches investigated the expression and biological function of S100A9 in NB. In the present study, the promoted expression levels of S100A9 were verified to be consistent with the microarray results. To further validate these results, our results suggested that elevated S100A9 promoted the proliferation and migration of NB cancer cells.

## Conclusions

In conclusion, the current observations indicate that S100A9 may be an important carcinogenic factor in the occurrence and progression of NB and may serve as a promising biomarker for metastasis prediction of NB patients. Nevertheless, well designed and multi-ethnics clinical researches with large sample will be necessary to verify and strengthen the metastatic role of S100A9 in NB patients.

## Data Availability

The following information was supplied regarding data availability: The data is available at NCBI GEO: GSE90121.
